# Heatstroke and brachycephalic dogs – is there an increased risk?

**DOI:** 10.18849/ve.v7i4.534

**Published:** 2022-12-22

**Authors:** Anna Ewers Clark+

**Keywords:** BOAS, BRACHYCEPHALIC OBSTRUCTIVE AIRWAY SYNDROME, BRACHYCEPHALY, CANINE, FLAT-FACED BREEDS, HEAT-RELATED ILLNESS, HEAT STRESS, HEAT STROKE, HRI

## Abstract

**PICO question:**

Do dogs that are brachycephalic have an increased risk for developing heat-related illness compared to dogs that are not brachycephalics?

**Clinical bottom line:**

**Category of research:**

Risk.

**Number and type of study designs reviewed:**

Four studies were critically appraised. Three of these were cohort studies, one was a case-control study.

**Strength of evidence:**

Moderate.

**Outcomes reported:**

Brachycephalic dogs are at increased risk of developing heat-related illness and brachycephalic breeds are over-represented in presentations for heatstroke. Other factors named, such as body weight, also contributed to the risk of developing heat-related illness.

**Conclusion:**

Dogs that are brachycephalic are likely to have an increased risk for developing heat-related illness compared to non-brachycephalic dogs.

## How to apply this evidence in practice

The application of evidence into practice should take into account multiple factors, not limited to: individual clinical expertise, patient’s circumstances and owners’ values, country, location or clinic where you work, the individual case in front of you, the availability of therapies and resources.

Knowledge Summaries are a resource to help reinforce or inform decision making. They do not override the responsibility or judgement of the practitioner to do what is best for the animal in their care.

## Clinical scenario

You are presented with a 3 year old male, neutered Pug with severe dyspnoea and tachypnoea. The day is sunny and 23^o^C, and the patient moderately exercised at midday. After stabilising and treating the patient, the owner wishes to discuss why their brachycephalic dog has needed emergency treatment when their Labrador was unaffected by exercise at the same time. You need evidence to explain that brachycephalic breeds can be more at risk of developing heatstroke.

## The evidence

The literature search revealed four papers relevant to the PICO. One study aimed to investigate risk factors for development of heat-related illness, another aimed to investigate the effect of brachycephaly on response to heat stress and the final two recorded brachycephaly of dogs as part of their aims to investigate heatstroke. The four papers provide evidence to support that brachycephalic dogs are at high risk or increased risk of heatstroke compared to non-brachycephalic dogs.

## Summary of the evidence

**Hall et al. (2020):** 

Population:	Client owned dogs with a record of presentation for heat-related illness (HRI) to UK VetCompass registered primary care veterinary practices from 2016–2018.
Sample size:	905,543 dogs, of which 1222 dogs experienced HRI (1259 HRI events).
Intervention details:	Database search carried out from submitted electronic patient records of primary care practices. Cases identified using final diagnosis, insurance claims, and / or clinical signs related to hot conditions (search parameters and inclusion criteria are clearly stated). Sample subdivided for separate analysis into ‘triggers for dogs with first HRI events’ and ‘fatality in HRI events between 2016–2018’. Risk factors for fatality and triggers analysed by multivariable logistic regression.
Study design:	Retrospective cohort study.
Outcome Studied:	Objective assessment of seasonality and event fatality for HRI and risk factor analysis for exertional, environmental, and vehicular HRI, (including breed type, skull shape, adult bodyweight, sex / neuter, and age).
Main Findings (relevant to PICO question):	Skull shape significant risk factor for HRI, with brachycephalic dogs having 1.32 times odds (95% CI 1.10–1.60, P = 0.004) of exertional HRI, 2.36 times odds (95% CI 1.50–3.72, P = <0.001) of environmental HRI and 3.07 times odds (95% CI 1.60–5.87, P = 001) of vehicular HRI compared to mesocephalic dogs. As individual breeds, Bulldogs and French Bulldogs have significantly higher odds ratio of environmental, exertional and vehicular HRI compared to Labrador Retrievers (Bulldog: environmental 7.52 OR, 95% CI 2.76-20.47, P = <0.01, exertional 3.73 OR, 95% CI 2.34-5.93, P = <0.01, vehicular 16.63 OR, 95% CI 3.02–91.53, P = 0.001; French Bulldog: environmental 3.16 OR, 95% CI 1.03–9.73, P =0.045, exertional 2.96 OR, 95% CI 1.96-4.47, P = <0.001, vehicular 6.70 OR, 95% CI 1.11–40.65, P = 0.039). Other brachycephalic breeds also significantly increased odds for some types of HRI. Majority of events presented due to exertional heatstroke (74.2%).
Limitations:	Unknown reliability of reporting from primary care veterinary practices may be subject to recall or observer bias. Potential for recording bias as some breeds more likely to be classified with HRI on presentation / with mild signs. Potential for bias due to missing data during data collection as retrospective study looking at clinical records. No record of prevalence of brachycephaly in population. Evaluated all presentations for HRI, using a wider definition of clinical signs. No standardised veterinary definition for heat-related illness or heatstroke. Brachycephaly classified according to breed type not individual patient skull morphology.

**Segev et al. (2015a):** 

Population:	Client owned dogs presented to the Emergency and Critical Care Unit of the Hebrew University Veterinary Teaching Hospital between 1999 and 2008.
Sample size:	126 dogs presented with heat-related illness (HRI).
Intervention details:	Clinical records searched for diagnosis of heatstroke which was based on clinical signs following exposure to heat stress. Clinical progression (from clinical records) and diagnostic test results evaluated. Variables analysed and weighted to create a predictive model for heatstroke with outcome. Model evaluated by logistic regression.
Study design:	Retrospective cohort study.
Outcome Studied:	Objective assessment of signalment, vital signs on presentation, history of exertional / environmental heatstroke, mortality, and results from diagnostic testing.
Main Findings (relevant to PICO question):	Brachycephalic breeds represented 36/126 (29%) of dogs in the study, which was higher than the general population presented to the hospital (16%). Brachycephalic breeds included Boxer, English Bulldog, French Bulldog, Dogue de Bordeaux, Chinese Shar-Pei, American Staffordshire Terrier, and Pekingese. No significant difference detected in mortality between brachycephalic and non-brachycephalic dogs. Majority of dogs had exertional heatstroke (64%).
Limitations:	No sample size calculation provided. Only recorded from one referral population, limiting generalisability. Dogs euthanised on financial grounds or where owner declined treatment were excluded, which may bias the included cases, for example those with less severe presentations perceived to have a better prognosis. Dogs with concurrent conditions excluded which may affect representation of some breeds (for example, some breeds are more likely to have breed-related health conditions). Unknown reliability of reporting from veterinary practice may be subject to recall or observer bias. Limited data available for classification of breeds. Full signalment data not available which means unable to compare brachycephalic breeds to other breeds presenting. No record of how brachycephaly of breeds was classified.

**Segev et al. (2015b):** 

Population:	Client owned dogs presented to the Emergency and Critical Care Unit of the Hebrew University Veterinary Teaching Hospital between 2011 and 2013.
Sample size:	30 individual dogs presented with heat-related illness (HRI) and 13 healthy staff owned dogs.
Intervention details:	Patients enrolled on diagnosis of heatstroke, inclusion criteria based on clinical signs and history. Blood samples analysed for markers of acute kidney injury at specified time points. Samples compared to healthy controls. Clinical outcomes of cases recorded and compared.
Study design:	Prospective cohort study.
Outcome Studied:	Objective assessment of signalment, clinical signs, renal biomarkers, and fatalities.
Main Findings (relevant to PICO question):	10/30 (33%) of enrolled dogs affected by HRI classified as brachycephalic. Of these dogs, most common breeds were Boxer and French Bulldog. Majority of dogs affected by environmental HRI; 10/30 (33%) affected by exertional HRI.
Limitations:	Client consent needed for enrolment so high likelihood of selection bias. Small sample size (and no sample size calculation provided). Method contains limited details so unable to tell if all patients received the same treatment or if laboratory sample analysis was blinded. Only recorded from one referral population, limiting generalisability. Dogs euthanised on financial grounds or where owner declined treatment were excluded, which may bias the included cases, for example those with less severe presentations perceived to have a better prognosis. Dogs with concurrent conditions excluded which may affect representation of some breeds (for example is some breeds are more likely to have breed-related health conditions). Unknown reliability of reporting as from veterinary practice- may be subject to recall or observer bias. No record of prevalence of brachycephaly in population for comparison. Limited data available for classification of breeds. Full signalment data not available which means unable to compare brachycephalic breeds to other breeds presenting. No record of how brachycephaly of breeds was classified.

**Davis et al. (2017):** 

Population:	Client owned dogs enrolled at Oklahoma State University.
Sample size:	105 dogs (52 brachycephalic).
Intervention details:	Dogs exposed to two environmental conditions using laboratory climate control system. Dogs were placed in a cool environment (to assess tolerance for measurements); followed by a hot environment (to assess response for heat stress). A room temperature maintenance phase was used between tests. Measures of heat stress were compared between brachycephalic and non-brachycephalic breeds (assigned based on breed type) and between hot and cold treatments.
Study design:	Case control study.
Outcome Studied:	Objective measures of respiratory pattern, body temperature, tidal volume, breathing cycle duration at different environmental condition.
Main Findings (relevant to PICO question):	5/52 (10.4%) brachycephalic dogs unable to complete hot environment trial due to respiratory distress. Respiratory rate increased significantly (P = 0.012) more in brachycephalic breeds than non-brachycephalic breeds when exposed to the hot environment. Brachycephalic dogs had a significantly (P = 0.004) greater increase in body temperature when exposed to the hot environment. However, this effect was only present when an interaction between breed type and body condition score was removed from the model. When this interaction was included, neither the interaction nor breed type was significant.
Limitations:	No sample size calculation provided. Evaluated response to laboratory controlled environment so may not be generalisable to naturally occurring HRI or heatstroke. All dogs exposed to cool temperature first and no consistent interval stated for testing meaning stress or other factors may have affected results. Although significance stated, no values for increase in body temperature or respiratory rate reported in results to determine if these are meaningful increases. No dogs with BOAS included. Brachycephaly assigned according to breed type not morphology.

## Appraisal, application and reflection

Heat-related illness (HRI) and heatstroke are serious and potentially fatal conditions in dogs (Bruchim et al., 2017).  The influence of breed and skull morphology are often discussed as risk factors for the development of HRI (Hall et al., 2020) and dogs with brachycephaly are often thought to be less able to cope with heat stress (Davis et al., 2017). This is supported by evidence appraised in this summary, which suggests that brachycephalic dogs are at increased risk of HRI compared to non-brachycephalic dogs.

The evidence provided by the summarised papers is moderate. Although there are no systemic reviews currently available for analysis, four studies were identified of relevance to the question: two retrospective cohort studies, one prospective cohort study, and one case-control study. Hall et al. (2020) has a large sample size and is likely to be representative for veterinary professionals in the UK as the study utilised clinical records from primary care practice. The study focused specifically on risk factors and development of HRI and identified skull shape as a significant factor for increased odds of exertional, environmental, and vehicular HRI, reporting that this was more significant than breed classification. However, other factors were also found to influence risk, including age and body weight. This was in line with the findings of Davis et al. (2017) which reported body condition score had a greater influence on response to heat stress than breed. It is important for this to be noted when making any recommendations with regards to breed risk for HRI, as the condition is often multifactorial (Hall et al., 2021).

Segev et al. (2015a) and Segev et al. (2015b) used alternative outcome measures, but they had smaller sample sizes and are less generalisable, recording findings from one referral population, and their findings include high numbers of brachycephalic dogs affected by HRI (36/126 [29%] of dogs in Segev et al. 2015a and 10/30 [33%] in Segev et al. 2015b). While Segev et al. (2015b) does not provide a comparison of prevalence of brachycephalic breeds within the wider population, the high percentage of dogs that were classified as brachycephalic in the study does suggest that they were commonly presented with HRI, and this is strengthened when contextualised alongside the findings of Segev et al. (2015a) and Hall et al. (2020) which contain comparisons showing an increased risk. Segev et al. (2015a) has stronger evidence for the increased risk in brachycephalic dogs as it provides a comparison of the percentage of brachycephalic dogs presenting with HRI, compared to the percentage presenting for other conditions at the hospital, making these results more generalisable to the wider population and reducing the chance of potential bias due to breed popularity.

In addition, Segev et al. (2015a) and Segev et al. (2015b) selected patients that presented with more severe HRI. The data has been collected from an emergency referral hospital which will be less likely to see mild cases of HRI compared to primary care practice, and the inclusion criteria selected for patients with severe clinical signs of HRI. The fact that brachycephlic dogs made up a high percentage of cases, and were shown to be over-represented compared to the general population in Segev et al. (2015a), points to brachycephalic dogs being at higher risk of clinically significant or severe HRI, although further evidence for direct comparison of severity of HRI in brachycephalic vs non-brachycephalic breeds would be needed to confirm this impact.

The findings from Davis et al. (2017) indicated that brachycephalic dogs were more sensitive to heat stress and experience a more profound thermoregulatory response, such as higher respiratory rates or development of respiratory distress, compared to non-brachycephalic dogs.

However, there are some limitations to interpretation of these studies.  It should be commented that there is a lack of standardised definitions for the condition and clinical signs of HRI, both between the papers and within the veterinary field. Many terms, including heatstroke, HRI and heat stress are used to describe the condition and the clinical signs of HRI can be non-specific, especially in the early stages. While scoring systems have been proposed (Segev et al., 2015a; and Hall et al., 2021) these have yet to be more widely utilised or validated. This weakens the strength of evidence and may lead to selection bias; especially as certain breeds may show more overt clinical signs related to HRI without the underlying pathology of significant non-pyrogenic hyperthermia. Further study and a validated scoring system for HRI events would enable stronger evidence for risk factors (including brachycephaly) for HRI to be generated.

The impact of the lack of a universal clinical definition or scoring for HRI is further compounded when looking at brachycephaly. Brachycephalic breeds may more frequently demonstrate the early clinical signs of HRI, such as panting, especially those that are suffering from Brachycephalic Obstructive Airway Syndrome (BOAS) (Packer et al., 2015), as they may be less tolerant of exercise (Lilja-Maula et al., 2017). This may increase the likelihood of a HRI diagnosis, based on their clinical signs alone without a meaningful increase in body temperature. Although Davis et al. (2017) attempted to address this with their ‘cool’ environment and found that in this environment there was no significant difference in the resting respiratory rates of brachycephalic and non-brachycephalic dogs, this study did not account for dogs that had been diagnosed with BOAS. Moreover, none of the other studies included BOAS as an independent factor for development of HRI, and several studies excluded dogs with concurrent conditions which may have included BOAS, so it is impossible to determine whether the effects of BOAS may have led to selection or interpretation bias. Further study to investigate the relationship of BOAS as a specific risk factor for HRI, including looking at the relationship between BOAS classification and severity of HRI, would help to determine if brachycephaly alone is a risk factor for HRI or whether BOAS is more relevant.

Some of the impact from the lack of definition for HRI can be mitigated when considering the inclusion and exclusion criteria in the studies. Hall et al. (2020) used a broad definition of heatstroke and included all recorded presentations classified as HRI, from mild to severe clinical signs. This means it is likely that the breadth of the different definitions for HRI were likely captured within these broad inclusion criteria. In addition, although the large number of observers (resulting from the use of primary care veterinary practice records) may have led to inconsistency, this does limit the chance of individual observer bias affecting the classification of HRI and means it is likely a more representative sample of the general UK dog population as primary care cases with HRI were included. Although this makes the study more generalisable, it is possible that these inclusion criteria may have selected for cases where HRI was not the true cause of clinical signs. However, the findings of increased brachycephalic risk for heatstroke were similar in Segev et al. (2015a) and Segev et al. (2015b) which had more specific inclusion criteria for dogs with more severe clinical signs of heatstroke.  Moreover, Davis et al. (2017) found that brachycephalic dogs developed hyperthermia more rapidly when exposed to heat stress, with significantly increased attempts at thermoregulation, compared to non-brachycephalic dogs. Although this study had methodology which used unnatural conditions compared to dogs presenting with HRI in veterinary practices, it does provide a standardisation of environment to ensure there is direct comparison between the different breeds and reducing the chance of differing environmental or exercise patterns being responsible for the increased risk.

A further limitation for consideration was that all the studies relied on breed classification to determine whether a dog was brachycephalic. Although Hall et al. (2020) identified skull shape as a risk factor to increase odds for HRI, the classification was based on recorded breeds and average skull morphology of the breed. The rest of the studies (Segev et al., 2015a; Segev et al., 2015b; and Davies et al., 2017) did not state their criteria for classification of breeds. No studies evaluated skull shape or degree of brachycephaly of the individual dogs studied as an independent variable to breed classification. There will be a wide variation of skull morphology present in dogs classified as a brachycephalic breed (Packer et al., 2015) especially as there will be variation in breed recording according to the observer, and not all animals classified as a particular breed will meet the breed standard for skull and muzzle conformation. This will especially impact the studies using clinical records to determine signalment. Further studies where skull to muzzle ratios are recorded would strengthen the evidence and reduce the risk of bias due to breed classification. However, some of this impact will be mitigated as it is thought that breed classifications tend to represent a minimum extreme for the breed (Knowler et al., 2019; and Packer et al., 2015), so it is likely that the evidence is still strong for brachycephalic dogs experiencing a higher risk of HRI.

It should also be noted that the studies analysed are from several different countries and include widely differing sample sizes, populations, and study designs. Although this means the results are not directly comparable, and therefore an overall prediction of odds or degree of increased risk is not possible, the population from the collective samples in these studies is felt to be generalisable to the population seen in veterinary practice, so provides meaningful evidence for a wide population of dogs.

The findings from these studies are felt to be highly relevant for dog welfare, especially given the current popularity of certain brachycephalic breeds such as French Bulldogs (O’Neill et al., 2018). Alongside this, there are concerns that skull and muzzle conformation are becoming more extreme in brachycephalic dogs (Knowler et al., 2019, Brachycephalic Working Group, 2020), which may further compound their risk of HRI. Raising awareness of the risk of HRI in brachycephalic dogs is important to help protect dogs from this potentially life-threatening condition. Efforts should be made to educate owners about the impacts exertional HRI (Segev et al., 2015a; and Hall et al., 2020) as well as the risk of environmental HRI, especially given the impacts of climate change which may lead to increased temperatures and extreme weather events in the UK and across the world (IPCC, 2022).

Overall, the studies provide moderate evidence to support brachycephaly as a risk factor in the development of HRI. Across all the cohort studies (Hall et al., 2020; Segev et al., 2015a; and Segev et al., 2015b), brachycephalic dogs were over-represented in presentations of HRI and in Davis et al. (2017), they were found to be more affected by exposure to heat stress. To provide stronger evidence, further study including systematic reviews with meta-analysis, impact of BOAS and specific investigations of skull morphology instead of breed classification would be beneficial. Additionally, it is also important to consider that HRI is a multifactorial condition and that other factors, such as body condition score and age, are also likely to be significant factors in the development of HRI in addition to skull morphology.

## Methodology

**Search Strategy:** 

Databases searched and dates covered:	CAB Abstracts on CAB Direct Platform (1914–Week 7 of 2022) PubMed on the NCBI Platform (1946–Week 7 of 2022)
Search strategy:	CAB Abstracts: heatstroke OR "heat stroke" OR "heat related illness" OR "nonpyrogenic hyperthermia" OR "heat stress" OR "heat strain" OR "heat induced illness" dog OR canine OR canid OR canis 1 AND 2 PubMed:((dog OR cani* OR canine) AND ("heatstroke" OR "heat related illness" OR "heat induced illness" OR "heat stroke" OR "non pyrogenic hyperthermia" OR "heat stress"))
Dates searches performed:	21 Feb 2022

**Exclusion / Inclusion Criteria:** 

Exclusion:	Articles irrelevant to PICO question. Textbook, case report or expert opinion. Studies not including brachycephalic breeds. Studies with study populations overlapping included study (most relevant study included). Studies investigating subgroups of heated related illness (HRI) presentations. Studies not recording proportional representation of brachycephalic breeds.
Inclusion:	Original research articles investigating HRI in dogs and including proportional representation of brachycephalic breeds.

**Search Outcome:** 

Database	Number of results	Excluded – Articles not relevant to PICO question	Excluded – Textbook, case report or expert opinion	Excluded – Studies not available in English	Excluded – Studies not including brachycephalic breeds	Excluded – Studies with study populations overlapping included study	Excluded – Studies investigating subgroups of HRI presentations	Excluded – Studies not recording proportional representation of brachycephalic breeds	Total relevant papers
CAB Abstracts	186	75	81	2	14	3	3	4	4
PubMed	183	139	25	1	2	6	3	3	4
Total relevant papers when duplicates removed									4

## ORCID

Anna Ewers Clark: https://orcid.org/0000-0001-6091-3109

### Conflict of Interest

Article prepared with assistance from University of Liverpool Veterinary Postgraduate Unit.
